# From Design to Closure: Artificial Intelligence Transforming Clinical Research

**DOI:** 10.7759/cureus.94895

**Published:** 2025-10-18

**Authors:** Kanika Vats, Mohammad Mazhar Alam

**Affiliations:** 1 Department of Research and Innovation, Emirates Classification Society (TASNEEF), Abu Dhabi, ARE; 2 Department of Laboratory, Disease Prevention Screening Center (DPSC), PureLab, Abu Dhabi, ARE

**Keywords:** artificial intelligence (ai), clinical research, clinical trial, data monitoring, digital health, machine learning, participant recruitment, safety monitoring, trial design, trial efficiency

## Abstract

Clinical research is essential as it advances medical innovation, from developing new treatments and improving existing ones for additional disease indications to creating better processes and the availability of medical devices, yet traditional trial methods are often slow, costly, and full of challenges. Over the past decade, the use of artificial intelligence (AI) and machine learning (ML) has evolved across all phases of the clinical research cycle, from study design and planning to initiation, conduct, and closure. This editorial explores how AI can create new opportunities to enhance patient recruitment, optimize trial design, improve dose adherence and participant retention, strengthen safety monitoring, and enable advanced data analysis. It also highlights key challenges associated with the use of AI/ML, including selection bias, privacy, ethical considerations, and regulatory compliance. Since these tools generate outputs based on trained datasets, issues like data drift must be carefully managed to ensure ongoing accuracy and reliability. By recognizing both opportunities and challenges of using AI/ML across all stages of clinical research, we have proposed potential solutions to help overcome these challenges and promote responsible adoption of this new technological era. Responsible deployment and rigorous validation are essential; although hybrid approaches combine AI-driven insights with human oversight, these technologies can improve trial efficiency, improve patient outcomes, and accelerate development of novel therapies, while ensuring that accountability, safety, and ethical integrity remain firmly with humans. This editorial provides a roadmap for integrating responsible use of AI into clinical trials, ensuring ethical integrity, regulatory alignment, and trust, so that AI ultimately strengthens trial outcomes and benefits the patients these studies are designed to serve.

## Editorial

Clinical trials remain the gold standard for driving medical progress, yet traditional approaches often encounter delays, high costs, and operational challenges. The growing use of artificial intelligence (AI) offers new opportunities to enhance trial design, improve patient recruitment, optimize monitoring, and improve safety. In parallel, regulatory frameworks are also evolving to ensure that AI-generated data can be trusted to support informed decisions on drug safety, efficacy, and quality.

AI refers to machine-based systems that operate under human-defined objectives to generate predictions, recommendations, or decisions that influence real or virtual environments. These systems typically gather inputs from both human and machine sources to understand context, process, and translate this information into models, and use these models to recommend actionable insights or recommendations. A commonly used subset of AI in drug development is machine learning (ML), which includes training algorithms to enhance their performance based on data-driven learning. While ML currently dominates the drug lifecycle, this editorial considers AI more broadly within the clinical research process.

This editorial examines how AI, with its evolving opportunities, can transform every stage of clinical research, from planning through trial closure, while addressing key challenges such as ethics, data privacy, and regulatory compliance, and offering potential solutions to overcome them.

Design and planning

The design and planning phase is crucial for the success of clinical trials, but many studies struggle due to poor patient selection strategies, recruitment challenges, and limited continual monitoring, even as research and development (R&D) investments continue to rise [1]. AI may accelerate data-driven decision-making by supporting patient identification, site feasibility assessment, and development of protocol design through analysis of available large databases in public databases, previous clinical research, and even from social media. For example, in ophthalmology trials, AI-driven recruitment has helped reduce costs and identify eligible patients most likely to benefit from emerging therapies.

ML models that have the ability to integrate clinical and genetic data allow more precise patient stratification and promote the adoption of personalized treatment strategies. Adaptive designs, such as AI-driven response-adaptive randomization (RAR), can optimize treatment allocation, improve response rates, and support in maintaining ethical standards. Advanced AI methods, including long short-term memory (LSTM) networks and neural ordinary differential equations, offer accurate modeling of pharmacokinetics and pharmacodynamics, helping predict optimal dosing and supporting personalized drug development. Apart from design and dosing, AI further enhances site performance, patient-site matching, data quality, diversity, operational efficiency, safety monitoring, and outcome evaluation [2].

Despite these opportunities, responsible AI use still demands careful consideration of data privacy, selection bias, human accountability, and informed consent. Early validation within the intended “context of use” is essential to ensure both reliability and compliance with regulatory requirements. 

Initiation

Patient recruitment continues to be a major challenge in clinical trials, creating a need for scalable and automated solutions. Automated solutions like AI-powered recommendation systems have the potential to match eligible patients to appropriate trials by analyzing both structured data, such as from electronic medical records (EMRs), and unstructured data, such as clinical notes and progress reports. When used via fine-tuned, open-source large language models (LLMs) within a retrieval-augmented generation concept, these systems support maintaining transparency, ensuring reproducibility, and feasible deployment, making them a suitable option for clinical settings.

In one of the oncology phase 1 trials, natural language processing (LMP) was used for patient-clinical trial matching, and the system achieved a precision of 73.68%, a sensitivity (recall) of 56%, an accuracy of 77.78%, and a specificity of 89.36%, demonstrating its usefulness, particularly for such biomarker-driven matches [3].

Moreover, such automated solutions can secure local deployment and standardize data formats (e.g., Phenopackets), improving recruitment efficiency and supporting advancements in precision medicine trials. However, careful implementation is needed to balance speed, safety, and equity.

Conduct

Patient adherence is crucial for maintaining trial integrity. Technologies that monitor medication adherence, guided by structured assessment criteria, can improve outcomes, while ML models help predict dropout risk and optimize patient engagement. AI-driven approaches can enhance recruitment, patient experience, and trial quality, but they must be implemented with rigorous validation and ethical oversight [4].

Wearable and digital health technologies are another support that allows continuous monitoring, early detection of adverse events, and supports personalized care. Smartphone-based digital biomarkers analyzed with AI support large-scale, noninvasive screening and risk assessment, further enhancing decision-making. However, algorithms trained on limited datasets may underperform across all age groups, ethnicities, or comorbidities. Many biomarkers lack validation in diverse populations, and sensor accuracy can vary by device or environment, emphasizing the need for care evaluation.

AI also has the potential to strengthen endpoint evaluation and safety monitoring. Social media monitoring can help early detection of adverse events, while neural network-based methods improve accuracy and consistency across data sources. Integrating standardized AI tools in a pharmacovigilance system could transform safety monitoring during trials and post-market surveillance.

However, it is strongly recommended that a hybrid approach combining AI-driven insights with human oversight be maintained to ensure that the technology supports, rather than replaces, clinical judgment.

Closure

Effective management of trial datasets is essential for ensuring completeness and supporting analyses. Hybrid strategies that combine ML-based imputation with prefilling improved data quality enable more accurate predictive modeling. AI-driven and in silico approaches further accelerate clinical research and reduce dependence on traditional experimental models, particularly in areas such as vaccine development.

Patient-specific digital twins combine mechanistic knowledge, clinical data, and AI to predict disease progression, guide individualized treatment, and improve preparedness for future healthcare challenges. Using historical data for prognostic covariate adjustment can further increase trial efficiency, reduce sample size requirements, and improve precision in treatment effect estimation [5].

**Figure 1:**
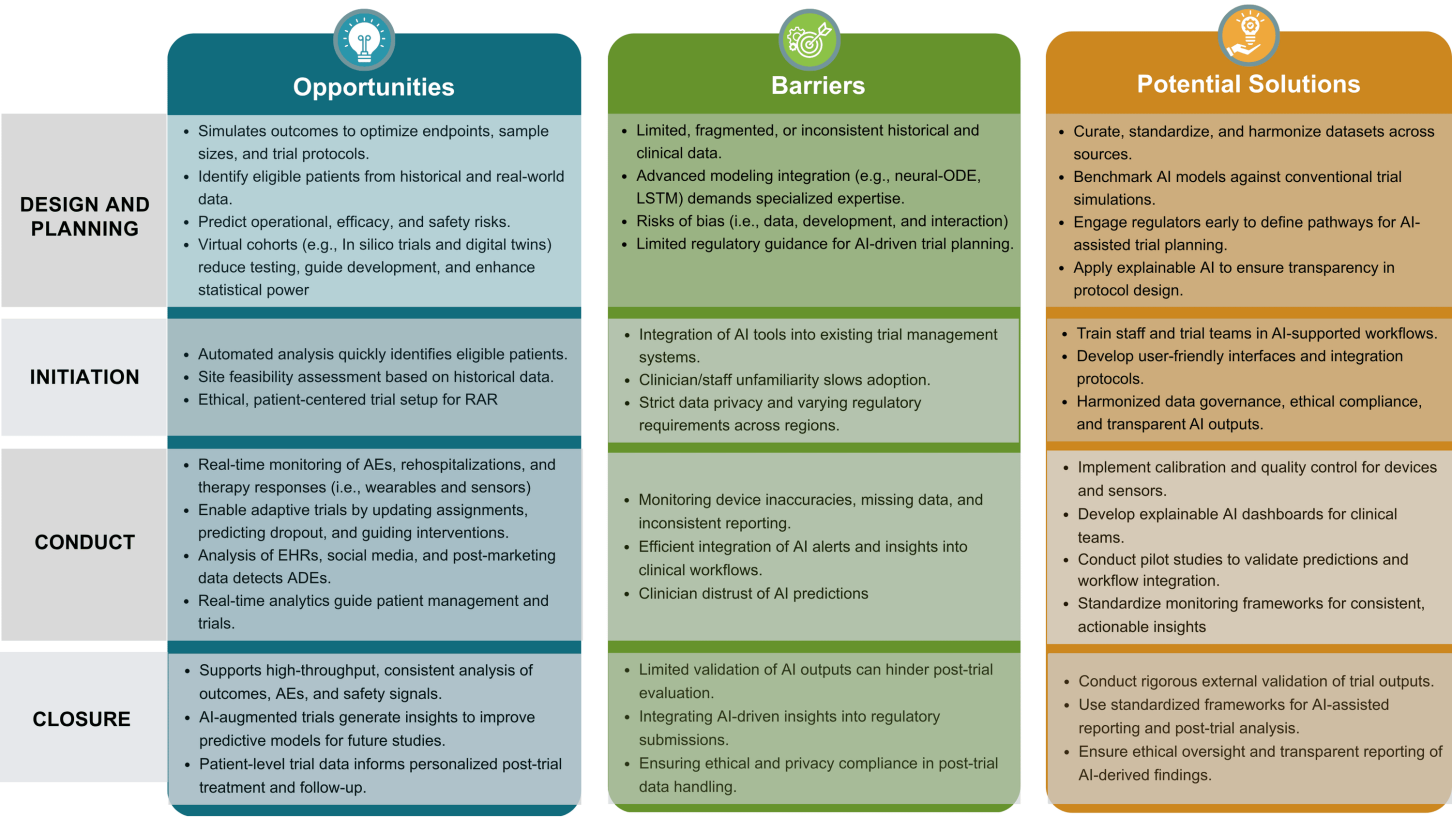
AI/ML applications across the clinical research cycle: opportunities, barriers, and solutions AEs: adverse events; ADE: adverse drug events; AI: artificial intelligence; EHRs: electronic health records; LSTM: long short-term memory; ML: machine learning; ODE: ordinary differential equations; RAR: response adaptive randomization The image is an original illustration created by the authors using the Canva platform (Canva Pty Ltd., Australia).

Figure 1 summarizes how AI/ML can enhance each stage of the clinical research cycle while highlighting barriers and practical solutions.

Responsible deployment of AI in clinical research requires collaboration among researchers, regulators, and technology developers. When guided by transparency, rigorous validation, and strong ethical safeguards, AI can make clinical research more efficient, patient-centered, and impactful, accelerating therapy development and advancing global health. However, this progress must be balanced with accountability and trust. In critical, life-to-death decisions, responsibility ultimately rests with humans, as machines cannot be held accountable in the same way, reinforcing the need for continuous human oversight at every stage of the clinical research cycle.

AL should serve as a tool, not a leader. While it can greatly enhance human capabilities, it must always operate under human oversight, ethical scrutiny, and regulatory control. Allowing machines to direct human life, particularly in sensitive areas like healthcare, is not only premature but potentially dangerous. The goal should be collaboration and not delegation.

In conclusion, AI holds transformative potential across the clinical research continuum. By enabling faster recruitment, more precise study designs, real-time safety monitoring, and efficient regulatory reporting, AI can support improving trial outcomes and patient care. However, realizing this potential requires careful attention to persistent challenges, including bias, privacy, and regulatory oversight to ensure that innovation remains safe, ethical, and human-centered. 
